# Cross talk between endothelial and red blood cell glycocalyces via near-field flow

**DOI:** 10.1016/j.bpj.2021.06.002

**Published:** 2021-06-29

**Authors:** Xi Zhuo Jiang, Michael S. Goligorsky, Kai H. Luo

**Affiliations:** 1 School of Mechanical Engineering and Automation, Northeastern University, Shenyang, Liaoning, China; 2 Department of Medicine Valhalla, New York; 3 Department of Pharmacology Valhalla, New York; 4 Department of Physiology, New York Medical College, Valhalla, New York; 5 Department of Mechanical Engineering, University College London, London, United Kingdom

## Abstract

Vascular endothelial cells and circulating red blood cell (RBC) surfaces are both covered by a layer of bushy glycocalyx. The interplay between these glycocalyx layers is hardly measurable and insufficiently understood. This study aims to investigate and qualify the possible interactions between the glycocalyces of RBCs and endothelial cells using mathematical modeling and numerical simulation. Dissipative particle dynamics (DPD) simulations are conducted to investigate the response of the endothelial glycocalyx (EG) to varying ambient conditions. A two-compartment model including EG and flow and a three-compartment model comprising EG, RBC glycocalyx, and flow are established. The two-compartment analysis shows that a relatively fast flow is associated with a predominantly bending motion of the EG, whereas oscillatory motions are predominant in a relatively slow flow. Results show that circulating RBCs cause the contactless deformation of EG. Its deformation is dependent on the chain layout, chain length, bending stiffness, RBC-to-EG distance, and RBC velocities. Specifically, shorter EG chains or RBC-to-EG distance leads to greater relative deflections of EG. Deformation of EG is enhanced when the EG chains are rarefied or RBCs move faster. The bending stiffness maintains stretching conformation of EG. Moreover, a compact EG chain layout and shedding EG chains disturb the neighboring flow field, causing disordered flow velocity distributions. In contrast, the movement of EG chains on RBC surfaces exerts a marginal driving force on RBCs. The DPD method is used for the first time, to our knowledge, in the three-compartment system to explore the cross talk between EG and RBC glycocalyx. This study suggests that RBCs drive the EG deformation via the near-field flow, whereas marginal propulsion of RBCs by the EG is observed. These new, to our knowledge, findings provide a new angle to understand the roles of glycocalyx in mechanotransduction and microvascular permeability and their perturbations under idealized pathophysiologic conditions associated with EG degradation.

## Significance

Vascular endothelial cells and circulating red blood cells are both covered by a layer of bushy glycocalyx. The interplay between these glycocalyx layers plays a pivotal role in maintaining vascular functionality/health but is hardly measurable and insufficiently understood. In this work, such interplay is interrogated via a computational method. Results suggest that the red blood cells drive the endothelial glycocalyx (EG) deformation via the near-field flow. Conditions that determine the interactions include length and density of EG chains, EG bending stiffness, and red blood cell velocity. By contrast, the propulsion of red blood cells by the EG is marginal. This study sheds additional light on the role of glycocalyx in mechanotransduction, microvascular permeability, and glycocalyx-related pathophysiologies under controlled, idealized conditions.

## Introduction

The endothelial glycocalyx (EG) is a delicate bushy structure interfacing blood and the endothelial cell membrane. Its hallmark functions include regulation of traffic of circulating proinflammatory cells, vascular permeability, mechanotransduction and signaling to adjust vessel diameter to the blood flow rate, and harboring distinct growth factors, regulators of coagulation, and extracellular antioxidant defense (1, 2, 3, 4, 5). The structural integrity of EG secures its functionality. Indeed, structural EG complexity is engendered in linear or globular proteoglycan core proteins decorated with covalently bound long chains of glycosaminoglycans. Together, they are responsible for the creation of the fine molecular fence, which performs as an external cellular sieve and depository for various bioactive molecules but also as sensory antennae of blood flow and communicational channels from the flowing blood. The spatial integrity of EG is paramount for both; however, it is frequently imperiled in disease states, as has been previously documented. Activation of heparanase, followed by pruning of heparan sulfate chains and accelerated degradation of EG in diverse pathological conditions, has been well established (6, 7, 8, 9, 10, 11).

EG is a carbohydrate-rich layer with highly negative charges. These charges empower EG to capture circulating plasma proteins and form an interconnected gel-like structure in an aqueous environment (12). The functional richness of EG is also encapsulated in its protruding spring-like molecular fence with an elastic modulus of ∼100–1000 Pa magnitude (13,14), which from the physical standpoint embodies a wealth of hidden information. Alas, direct approaches for dynamic in situ experimental interrogation of this delicate nanostructure are nonexistent. Therefore, it is anticipated that numerical modeling of EG may circumvent this deficiency and offer insights into its intrinsic physical properties and behaviors with the passage of blood with red blood cells (RBCs). The latter is especially relevant to the pathophysiology of microcirculation in the light of Oberleithner’s studies on “fingerprinting” between endothelial and RBC glycocalyx (15). To be specific, a vicious cycle could ensue when either RBC or endothelial cell surface layers are somehow damaged, resulting in reciprocal pathologic changes of glycocalyces.

Numerical modeling has played an increasingly important role in understanding EG functionality over the last three decades. Initially, continuum modeling of EG was conducted with a limited number of glycocalyx features. For example, the glycocalyx was simplified as a cylinder (16) and the EG layer as a porous medium (17). The recent progress and wide application of supercomputers enable numerical modeling to resolve far more complex EG-related problems with high-resolution outputs that wet-lab experiments are unable to achieve. For example, complex continuum models were constructed to demonstrate that ion-EG interactions can influence the local flow (18). High-resolution atomic-scale methods such as the molecular dynamics (MD) method, though computationally expensive, have also started to be applied to numerical modeling of EG (19, 20, 21, 22). We have recently employed MD to probe details of flow over EG (19,20), modes of glycocalyx-mediated mechanotransduction (21), and the mechanism of microvascular ion transport (22). Several remaining questions are related to the contrasting flow patterns in compact and loose EG layouts: the behavior of individual heparan sulfate (HS) chains under flow conditions, the contribution of shortened HS chains to such a behavior, the near-contact interaction of the EG and RBC glycocalyx layers facing each other, and the effects of the shortened HS chains on the above interactions. MD is a powerful tool to capture key events occurring on the EG surface but at a high computational cost, thus limiting the extent of observed individual molecules and duration of observations. Alternatively, dissipative particle dynamics (DPD) has proven capabilities for capturing the dynamic and rheological properties of simple and complex fluids, and thus may be deployed to solve the proposed questions at an acceptable accuracy and an affordable computational cost. So far DPD has been successfully applied to investigate biomolecular behavior, such as DNA moving in nanoslits (23) and endocytosis of liposomes (24). In this study, we use DPD to gain insight into the interactions between the EG and the glycocalyx of passaging RBCs. By doing so, we hope to answer the above proposed questions and understand the mechanisms of glycocalyx-dependent mechanotransduction in the EG and the ensuing microcirculatory disturbances when the EG is degraded.

## Materials and methods

### DPD

DPD is a mesoscale method for understanding the hydrodynamics of soft matter and complex fluids via coarse-grained models (25,26). In the basic form of DPD, the particles interact via pairwise additive forces, consisting of three terms: a conservative force **F**^C^, a dissipative force **F**^D^, and a random force **F**^R^. Thus, the total force on particle *i* is given by

(1) $$F_{i}=\sum_{i\neq j}F_{ij}^{C}+F_{ij}^{D}+F_{ij}^{R},$$

where the sum acts over all particles within a cutoff radius *r*_c_. The individual terms on the right-hand side are given by

(2) $$\begin{array}{l} F_{ij}^{C}=a_{ij}w\left(r_{ij}\right)e_{ij}\\ F_{ij}^{D}=-\beta_{ij}w^{2}\left(r_{ij}\right)\left(e_{ij}\cdot v_{ij}\right)e_{ij}\\ F_{ij}^{R}=\sqrt{2\beta_{ij}k_{B}T/\Delta t}w\left(r_{ij}\right)\alpha e_{ij}, \end{array}$$

where *r*_*ij*_ is the distance between two particles *i* and *j*, **e**_*ij*_ is the unit vector from particle *i* to *j*, and *a*_*ij*_ represents the maximal repulsive force. In this study, the maximal repulsive force between a glycocalyx bead (g) and a water bead (w) is *a*_gw_ = 26.3, the glycocalyx-glycocalyx repulsive force is *a*_gg_ = 30, and the water-water force is *a*_ww_ = 25 (26). *w*(*r*_*ij*_) is a weighting factor following *w*(*r*_*ij*_) = 1 − *r*_*ij*_/*r*_c_ at *r*_*ij*_ ≤ *r*_c_ and *w*(*r*_*ij*_) = 0 at *r*_*ij*_ > *r*_c_. *β*_*ij*_ is the friction coefficient relying on the value of *a*_*ij*_. In this study, *β*_*ij*_ is set to be 4.5. In the random force term, *k*_B_ is the Boltzmann constant, *T* is the thermodynamic temperature, *α* is a Gaussian random number with zero mean and unit variance, and Δ*t* is the timestep size. The time step used in this study is Δ*t* = 0.001*τ* (*τ* is the DPD time unit and will be mentioned later).

### Glycocalyx model

A glycocalyx unit comprises glycosaminoglycans (GAGs), proteoglycans, and other glycoproteins. Among them, GAGs are exposed to blood, and their physical positions empower them to actively interact with other cells. HS is a type of GAG chain predominantly bound to the distal portion of core proteins and accounting for ∼50–90% of the GAG pool (27). As this study focuses on the cell interactions, a model of HS chains was constructed as a simplification of glycocalyx. An HS chain was simulated by a bead-string chain that contained 60 beads. The chosen number was based on the length scale of an HS chain (28,29). Adjacent beads comprising the glycocalyx chains are connected by a harmonic spring with potential in the form of

(3) $$E_{\mathrm{bond}}=k_{s}{\left(r_{ij}-b_{0}\right)}^{2},$$

where *k*_s_ = 100 *k*_B_*T*/*r*_c_^2^ is the spring coefficient, and *b*_0_ = 0.5 *r*_c_ is the equilibrium bond length distance. The bending energy was given by

(4) $$E_{\text{bend}}=1/2k_{\text{E}}{\left(\varphi -\varphi_{0}\right)}^{2},$$

where *φ* is the angle among three neighboring particles and *φ*_0_ = *π* is the equilibrium angle; the harmonic bending constant *k*_E_ and the bending stiffness (*EI*) of the glycocalyx are related by the Euler-Bernoulli beam theory in the form of *EI* = *k*_E_ × *b*_0_. *EI* for the EG is ∼490 pN nm (30).

### Case setups

**Figure 1:**
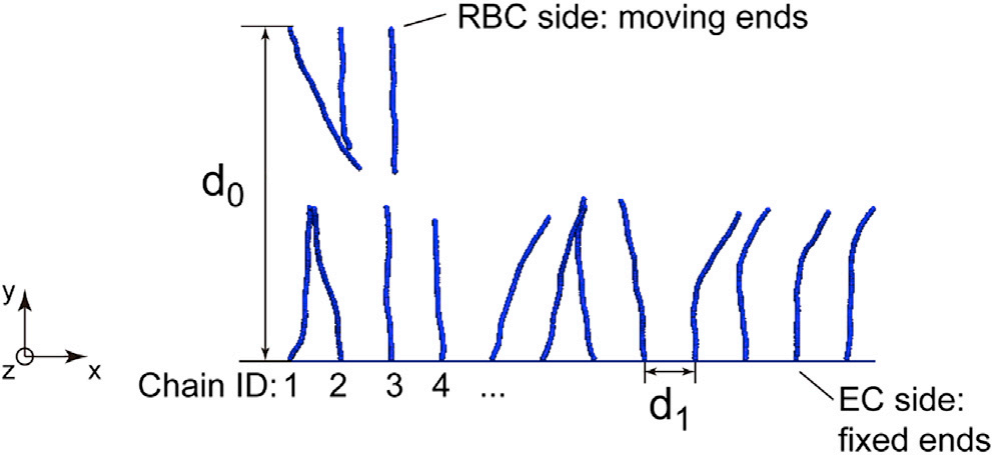
DPD model for studying RBC glycocalyx brushing EG. The upper bead chains represent RBC glycocalyx, and the lower array of chains represent glycocalyx on the endothelial cell (EC) surface. For clarity, water beads are not shown. To see this figure in color, go online.

A two-compartment model with EG chains driven by flow was first investigated in case 0 to compare the DPD method with our previous MD method. In case 0, external forces were imposed on water beads to mimic flow, and the dynamics of EG chains were monitored. Then, three-compartment models were built to reveal the interactions between the RBC glycocalyx and the EG. In the three-compartment models, the main purpose is to test whether RBC glycocalyx and EG can propel each other; for such a purpose, RBC glycocalyx and EG were solvated in water beads, but no external forces were imposed on water beads. To mimic the near brushing of the RBC glycocalyx over the EG, two arrays of glycocalyx chains were generated as shown in Fig. 1. The EG chains were represented by the lower array in which semiflexible chains were tethered to the wall. The upper array was constructed to represent the moving RBC glycocalyx. To mimic the movement of the RBC glycocalyx, a constant velocity in the *x* direction was imposed on the upper ends of chains.

The distance between the far ends of the two arrays was labeled by *d*_0_, and the distance between fixed ends of adjacent chains was labeled by *d*_1_. By varying *d*_0_ and *d*_1_, the influence of the RBC-EC distance and EG density on glycocalyx dynamics can be investigated. By changing the number of beads in a chain and *k*_E_ in Eq. 3, the impact of EG length and bending stiffness, respectively, can also be studied, thus allowing better emulation of pathologic conditions with degraded glycocalyces. The influence of RBC moving velocity on the behavior of EG chains is also included by analyzing cases A1 and A2, in which the RBC velocities are varied. To investigate how the shedding of RBC glycocalyx affects EG deformation, case A3 is designed, in which the number of RBC glycocalyx chains is reduced to one and the setting for EG is exactly the same as that in case A. The case setups in this study are summarized in Table 1. Case G is an extension of case A in which the influence of a shedding chain is dissected (detailed in EG deformation induced by the propulsion from RBC glycocalyx). Case H is generated to scrutinize the response of RBCs to the EG motions (detailed in EG as a propulsor for RBC movement). It should be mentioned that in reality, RBCs could have tumbling or tank-treading motion that causes glycocalyx translation against the direction of flow. The proposed three-dimensional model can be extended to include such rotational motions, which would require a much larger computational domain and significantly increase computational cost. The simulation domain of DPD cases in Table 1 is 80 × 45 × 40 *r*_c_^3^. Every individual system was first equilibrated for 200*τ*, and afterwards a velocity of 0.05 *r*_c_/*τ* was imposed on the moving ends of the RBC glycocalyx. Generally, the production simulation lasted for 1600*τ*. Periodic boundary conditions were applied to the *x* and *z* directions. In the *y* direction, a wall restriction was set for the lower chains, and the wall interacted with the surrounding beads by generating a force on the beads in a direction perpendicular to the wall. The average particle number density of the DPD system is 3.0*r*_c_^−3^, and the temperature is set to *k*_B_*T* = 1. In line with the HS chain length (∼30 nm (29)) and the order of RBC velocities (31), *r*_c_ = 0.5 nm and the order of *τ* is ∼0.01–0.1 *μ*s. Accordingly, one DPD force unit is ∼1–10 pN in this study.

**Table 1:** Cases used in this study

Case	*d*_0_ (*r*_c_)	*d*_1_ (*r*_c_)	Number of EC chains	Number of beads of an EC chain	*k*_E_/2	Remarks
0	N/A	5	12	60	231	EG in flow
A	32.5	5	12	60	231	normal condition
A1	32.5	5	12	60	231	slow RBCs
A2	32.5	5	12	60	231	fast RBCs
A3	32.5	5	12	60	231	RBC glycocalyx shedding
B	32.5	2.5	24	60	231	dense EG
C	32.5	10	6	60	231	rarefied EG
D	32.5	5	12	30	231	short chains
E1	32.5	5	12	60	347	stiffer EG
E2	32.5	5	12	60	115	softer EG
E3	32.5	5	12	60	462	stiffer EG
E4	32.5	5	12	60	693	stiffer EG
F	27.5	5	12	60	231	close RBC-EG
G	32.5	5	12	60	231	release chain 7
H	32.5	5	5	60	231	EG propels RBC

EC, endothelial cell; N/A, not applicable.

All DPD simulations were performed using Large-scale Atomic/Molecular Massively Parallel Simulator (LAMMPS) (32). The visualization of the structures was performed via the Visual Molecular Dynamics (VMD) (33) package. All parallel simulations and nonvisualized postprocessing were conducted on ARCHER2, the UK’s new-generation national supercomputing service.

### Statistical analyses

Data were sampled every DPD time step. The sample sizes of case 0 and E1–E4 are 400, case G 800, and others 1600. Unless otherwise indicated, data are presented in the form of mean ± standard error. Tests of null hypotheses vary, depending on the aims, which are mentioned individually. Statistical tests were conducted using SciPy, a Python-based open-source scientific computing tool (34).

## Results

### EG deformation induced by flow: a two-compartment model

Our previous MD simulations of EG and flow (19,20) showed dependence of flow patterns on the structure of EG and “swing and swirling” patterns of the glycocalyx itself. When the case (case 0) is simulated by DPD in this study, bending of EG chains toward the direction of flow emerge. When the flow was slow, EG chains oscillated as they bent; when the flow accelerated, the bending became dominant (see Video S1), and the chains tended to bend in a synchronized manner. These findings are consistent with the spring-like model of EG and prompt the questions of opposing EG and RBC glycocalyces, as described below.

Video S1. Flow by a two-compartment model

### EG deformation induced by the propulsion from RBC glycocalyx

**Figure 2:**
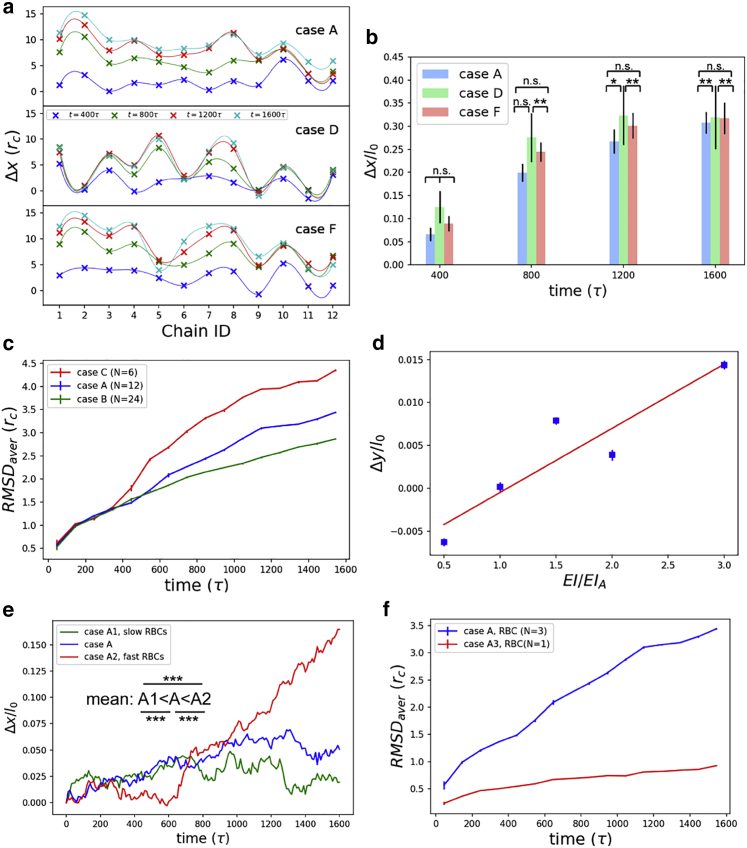
Deformations of EG chains are subjected to chain length, RBC-EG distance, chain density, bending stiffness, and RBC velocities. (*a*) Individual chain *x* deflection in cases A, D, and F. Case A represents the “normal” condition; in case D, the EG chains are halved in length; RBC and EG are closer in case F. The cross markers are simulation results, and the smooth curves are constructed by B-spline curves. Dynamics of EG chains in cases A and D can be found in Video S2. (*b*) The relative *x* deflection in cases A, D, and F. (*c*) Average RMSDs of EG chains under varying chain density situations. Dynamics of EG chains in cases A, B, and C can be found in Video S3. (*d*) Bending of EG chains as a function of glycocalyx bending stiffness (*EI*). *EI*_A_ is the bending stiffness of case A. The square markers are simulation results, and the line is from linear regression. (*e*) Influence of RBC driving velocities on EG chain deformation in terms of relative *x* deflection. The significance was tested by *t* tests. ^∗∗∗^*p* < 0.001, ^∗∗^*p* < 0.01, ^∗^*p* < 0.05, and n.s. for “not significant.” Dynamics of EG chains under varying RBC velocities can be found in Video S4. (*f*) Average RMSDs of EG chains under normal and RBC-chain-shedding cases. Dynamics of EG chains in both situations are provided in Video S5. To see this figure in color, go online.

Theoretically, the RBC glycocalyx may cause the deformation of EG chains via the near-field flow field and flow shear stress. The RBC glycocalyx chains first drive the movement of water beads, and water beads, then transmit the momentum to the glycocalyx chains on the endothelial cell side, causing further deformation of EG chains. Fig. 2 illustrates the influence of chain length, RBC-EC distance, chain density, and bending stiffness on the deformation of EG chains.

#### Spatial determinants: chain length and RBC-EG distance-dependent deflection

The *x* deflections of individual chains were compared among cases A, D, and F. As shown in Fig. 2 *a*, as RBC glycocalyx chains move forward and continuously drive the movement of water beads, the deflections of EG chains (Δ*x*) increase with time. Under the resistance from the bending stiffness, the EG chains will eventually achieve new equilibrated positions. In case D, in which the EG chain length was halved compared with case A (a “normal” situation), the EG chains reached their new equilibrated positions quickly. (Dynamics of chains in cases A and D are shown in Video S2.) To illustrate the EG-flow interface, B-spline curves were used to construct the surface formed by the EG free ends. A B-spline curve is a type of nonlinear interpolation that facilitates the construction of complex interfaces. By regarding the EG chains as cantilever beams, the time of a cantilever beam to reach its first maximal deflection *t* is inverse to the vibration frequency *f* (i.e., *t* ∼*f* ^−1^). Incorporation of the relationship between vibration frequency *f* and beam length *l*_0_ (*f* ∼*l*_0_^−2^) then gives *t* ∼*l*_0_^2^. Thus, the short chains in case D reach equilibrated positions earlier than their long counterparts in case A. The short time to achieve new equilibrium in case D suggests that shorter chains are susceptible to flows. In case F, in which the RBC and EG were closer to each other, the shorter distance between the RBC and EG arrays allowed stronger interactions among chains and thereby changed the chain deflections. Considering the EG chain lengths were different among cases A, D, and F, relative deflection Δ*x*/*l*_0_ was averaged among all the EG chains. As shown in Fig. 2 *b*, larger relative deflection occurred in the short-chain case (case D), and the closer distance between the RBC and endothelial cell enhanced the interactions among glycocalyx chains and thereby the relative deflection (case F). These findings corroborate the prediction that the EG and RBC glycocalyx interact with each other via the near-field flow field as mentioned at the beginning of EG deformation induced by the propulsion from RBC glycocalyx.

Video S2. Effects of chain lengths on EG deformation

#### Deformation and chain density

The deformation of chains is quantified by the average root mean-square deviation (RMSD) defined by

(5) $$\text{RMSD}={\left(\frac{{\sum_{i=1}^{N_{\text{b}}}w_{i}\left\|x_{i}-y_{i}\right\|}^{2}}{N_{\text{b}}\sum_{i=1}^{N}w_{i}}\right)}^{1/2},$$

where **x** and **y** are two consecutive structures of EG chains, *N*_b_ is the number of EG chain beads, and *w*_*i*_ is a weight coefficient and identical among atoms in this study. The RMSD was averaged every 100*τ* for cases A, B, and C in which the EG densities varied. As shown in Fig. 2 *c*, the average RMSD value increased as the number of EG chains decreased. (Dynamics of chains in cases A, B, and C are shown in Video S3.) The less compact layout of EG chains in case C increased the freedom for the chains to move, which accounted for the larger RMSD value; by contrast, chains in case B were confined because of the compact layout, thereby resulting in a smaller deformation. Such results are consistent with our previous conclusions from MD simulations (21). The trend of RMSD with the number of EG chains further implies that chains are self- and neighbor-protective by preventing severe deformation of each other when their density is optimal, but lose this quality when chains are rarefied.

Video S3. Effects of chain layouts on EG deformation

#### Stretching and bending stiffness

To reveal the role of mechanical properties of chains in EG deformation, deformations of EG chains with varying *EI*-values were compared. For a fair comparison, the same conformation of chains was used as the initial input for cases A and E1–E4. The conformation was extracted from the last frame of the 200*τ* equilibrium simulation, in which the value of *k*_E_/2 for *EI* was 231. Afterwards, the RBC glycocalyx drove the EG with varied *EI*-values. Therefore, the EG chains could either bend or stretch when the RBC passes by, which depends on the mechanical properties of the chains. As most of cases E1–E4 had greater *EI*-values, we used “stretching” to describe the further deformation. The stretching of chains was gauged by the relative height changes of tip beads (Δ*y*/*l*_0_) in five cases with varying bending stiffness (cases A, E1, E2, E3, and E4). Notably, the four bending stiffnesses in cases E1–E4 are not necessarily realistic but used mainly to reveal the trend. The relative height change as a function of relative bending stiffness is illustrated in Fig. 2 *d*. The increasing trend suggests that a greater bending stiffness facilitates the maintenance of the stretching conformation of chains, as stiffer chains resist against bending deformation.

#### RBC velocity and chain bending

The influence of RBC velocity on EG chain deformation was implemented by varying the driving velocities of the moving ends of the glycocalyx on the RBC side. By changing the velocity of the moving ends to 0.005 *r*_c_/*τ*, a slow RBC situation was simulated (case A1), and in the fast RBC case (case A2), the velocity of the moving ends was set to be 0.2 *r*_c_/*τ*. To mitigate the potential influence of the periodic boundary conditions in the fast RBC case (some beads may move out of the box and enter the other side of the box because of the fast velocity), the relative deflection was calculated for the central two chains (i.e., chains 6 and 7). The time evolution for the relative deflection (Fig. 2 *e*) shows that the fast movement of the RBCs caused severe bending of the EG chains. (The dynamics of chains in cases A, A1, and A2 are shown in Video S4.)

Video S4. Effects of RBC velocities on EG deformation

#### Shedding RBC glycocalyx and EG deformation

To investigate the impact of RBC glycocalyx shedding on EG behavior, deformation of EG was compared between normal (case A) and RBC-glycocalyx-shedding (case A3) situations. RMSD values were calculated to quantify the EG deformation, as shown in Fig. 2 *f*. Results show that case A3 has a smaller RMSD than its case A counterpart, which suggests a weaker response of the EG to the flow driven by the RBC glycocalyx. (Dynamics of EG chains in cases A and A3 are demonstrated in Video S5.)

Video S5. Effects of RBC glycocalyx shedding on EG deformation

The deformation of EG chains is a consequence of forces exerted therein, and the forces are the actual form of mechanical signals transmitted by EG. Thus, results obtained from this section have implications for the role of EG in mediating mechanotransduction. Detailed analysis is given the Discussion.

### Flow patterns

#### Flow order or disorder and chain density

**Figure 3:**
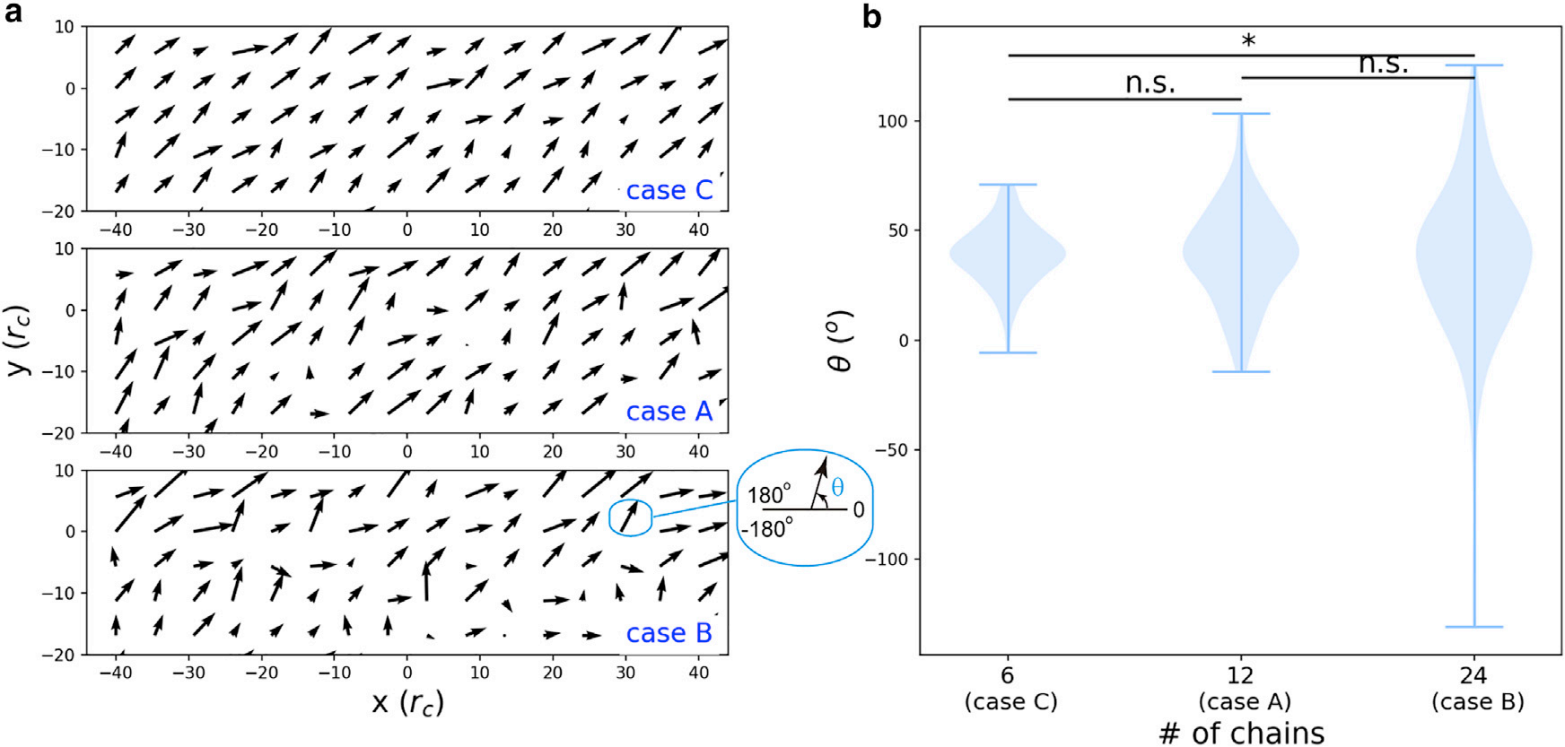
Disturbance in flow field by the chain density. (*a*) Flow velocity distribution in the presence of varying glycocalyx chain numbers. Note that a less compact glycocalyx layout is associated with reduced chaos in the flow field. Remarkably, the general orientation of the flow is in the upward direction, tending to maintain the spatial separation of glycocalyces of endothelial cells and RBCs. (*b*) Comparisons of flow orderliness among cases C, A, and B. The orderliness is quantified by distributions of flow velocity orientation. A Kolmogorov-Smirnov test was used to test the null hypothesis with ^∗^ meaning *p* < 0.05. To see this figure in color, go online.

The RBC drives the EG mainly via the near-field flow, as they do not physically contact each other. The flow pattern was then scrutinized in terms of the velocity distribution. The whole simulation domain was equally divided into 16 × 9 × 1 cuboid meshes, and the lengths of side of individual meshes in the *x*, *y*, and *z* directions are 5 *r*_c_, 5 *r*_c_, and 40 *r*_c_, respectively. The velocities of beads in an individual mesh were then averaged for cases A, B, and C in which the number of EG chains varies. Fig. 3 shows the velocity projection on the *x-y* plane. The length of an arrow represents the velocity projection magnitude, and the orientation is the direction. As shown in Fig. 3 *a*, the flow velocities in case C with 6 EG chains tend to move in the same direction; by contrast, as the number of EG chains increases, the flow becomes chaotic as in cases A and B. To quantify the order or disorder of the flow velocities, the orientation of each arrow was calculated (labeled by *θ*), and the orientation distribution was compared. As shown in Fig. 3 *b*, the angle variation increases as the chain number increases, which implies that the movement of EG chains would generate chaos in flow. Notably, in all three cases the resulting flow vectors have an upward orientation suggestive of a “lifting force” (35), which secures the formation of the cell-free layer between the RBCs and the blood vessel walls.

#### Flow orientation and shedding of EG chains

**Figure 4:**
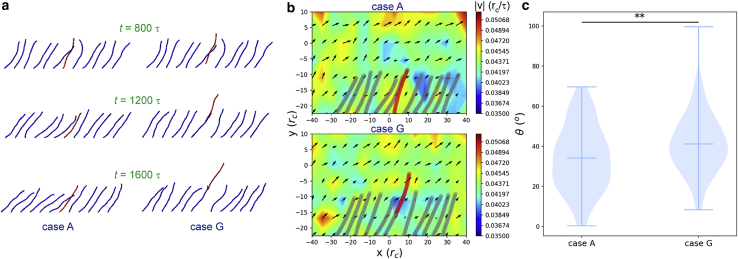
Influence of a shedding chain on the flow field. (*a*) Comparisons of EG chain morphologies with (case G) and without (case A) a shedding chain. Video S6 is also provided to show the dynamics. (*b*) Flow velocity distributions for cases A and G, respectively. The velocities and positions of chains are averaged over the last 400*τ*. The color bar represents the velocity magnitude. (*c*) Comparison of average flow velocity orientation between cases A and G. A Kolmogorov-Smirnov test was used to test the null hypothesis with ^∗∗^ meaning *p* < 0.01. To see this figure in color, go online.

Degradation of the EG and shedding of HS chains are attributes of diverse pathological conditions affecting cell and organ functions (36, 37, 38). To reveal the potential impact of a shedding chain on the flow field, case G was designed: at *t* = 800*τ* in case A, a central chain (ID 7) was displaced upwards by 2*r*_c_ in the +*y* direction, and the restriction on the fixed end of chain 7 was removed; the simulation then lasted for another 800*τ*. Fig. 4 *a* and Video S6 illustrate the dynamics of chain 7 after being released in case G; the chain gradually moved upwards. The upward momentum was then transmitted to flow, disturbing the flow field (Fig. 4 *b*) and causing an increase in *θ* (*bar* inside each *violin plot* of Fig. 4 *c*). Also, a larger variation of *θ* (range of each *violin plot* of Fig. 4 *c*) was observed in the shedding-chain case (case G), which means the flow is more disordered. The *y* direction forces of individual beads on chain 7 were recorded. The average *y* direction force on a single bead was 0.2 DPD force, indicating the lifting force from water is of an order of ∼0.1–1 pN, which agrees with previous calculations (35).

Video S6. Shedding of Chain 7

Being a conveyor of blood components throughout the body, blood vessel flow carries out the important function of mass exchange to support metabolic homeostasis. The findings reported in this section reveal how the shedding of EG chains alters the flow field. The alteration in the flow field then affects the motion of blood components such as ions and further changes their permeability (see Discussion for details).

### EG as a propulsor for RBC movement

In EG deformation induced by the propulsion from RBC glycocalyx, we demonstrated that the RBC drives the motion of the EG via the near-field flow field, and EG chains respond differently as the distance between RBC-EG and RBC velocities change. Here, the inverse question—could the motion of EG chains, in turn, propel the RBCs?—is proposed and resolved.

**Figure 5:**
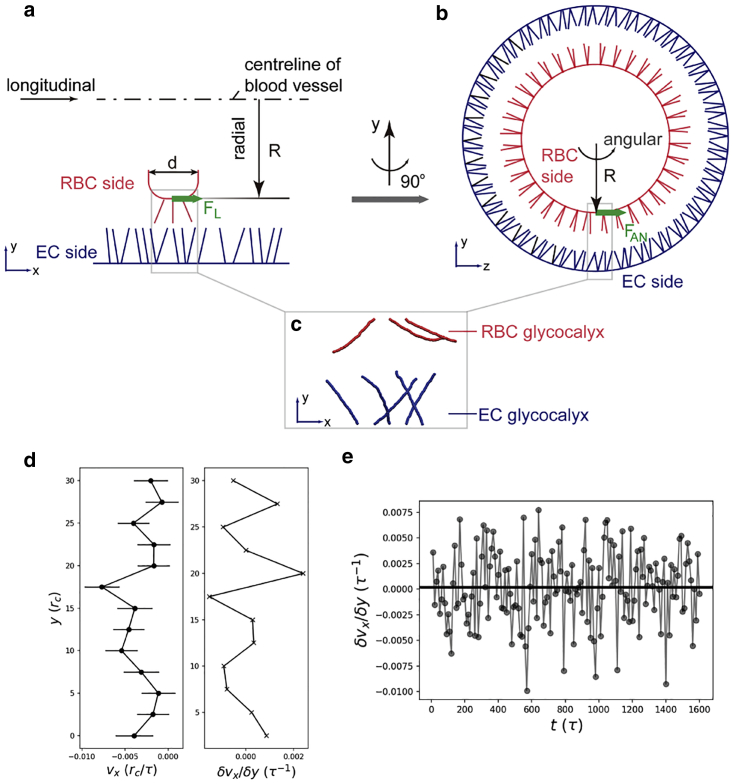
Schematic of RBC glycocalyx and EG. (*a*) Longitudinal cross section. Only a part of the RBC edge is shown. (*b*) Radial cross section. (*c*) Snapshot of DPD simulations of the glycocalyx on the RBC and endothelial cell (EC) sides. (*d*) Velocity (*left*) and shear rate (*right*) distributions for the last 400*τ*. (*e*) Time evolution for shear rates in the region (*y* = ∼25*r*_c_–30*r*_c_ in *d*) near the RBC surface. Gray dots are instantaneous shear rates, and black solid line is the average value of the shear rate. The shear rate is fluctuating around zero (the average is 0.0002/*τ*), and the shear stress on the RBC surface is miniscule. To see this figure in color, go online.

Fig. 5*, a and b* illustrate the spatial layout of glycocalyx chains on the RBC and the endothelial cell sides. Theoretically, the movement of the EG could drive the motion of RBCs in both the longitudinal and angular directions via the near-field flow field, like what the RBC glycocalyx does to the EG chains. In this section, a new DPD simulation was constructed. Beforehand, several imposed assumptions should be clarified.

To facilitate analysis, we assumed that the glycocalyx is axisymmetric along the centreline of the blood vessel. (The influence of asymmetry will be discussed later.) Under such an assumption, in the longitudinal direction (Fig. 5 *a*), the driving force *F*_L_ is from the wall shear stress of the flow (*f*_L_) induced by the motion of glycocalyx on the endothelial cell side and *F*_L_ = *f*_L_ × *A*. *A* is the area of the RBC outline. For simplicity, an RBC is assumed to be represented by a cylinder with a radius of *R* and thickness of *d*, which gives *A* = *πR*^2^*d.* According to Newton’s Law of Motion, the acceleration of the RBC (*a*_RBC_) is then calculated as

(6) $$a_{\text{RBC}}=F_{\text{L}}\text{/}m_{\text{RBC}}=f_{\text{L}}\times A\text{/}m_{\text{RBC}},$$

where *m*_RBC_ is the mass of an RBC. The only unknown in Eq. 6 is the wall shear stress which will be obtained from the following DPD simulation results of case H.

Analogously, as illustrated in Fig. 5 *b*, the angular force (*F*_AN_) from the wall shear stress (*f*_AN_) drives the angular motion of the RBC cell. The angular velocity of the RBC (*w*_RBC_) is calculated by the moment of momentum (**M**),

(7) $$\begin{array}{l} M=R\times F_{\mathrm{AN}}=J_{L}w_{\mathrm{AN}}\\ \Rightarrow w_{\mathrm{AN}}=J_{L}/\left(R\times F_{\mathrm{AN}}\right)\\ \Rightarrow w_{\mathrm{AN}}=J_{L}/\left(R\times f_{\mathrm{AN}}\times A\right), \end{array}$$

where *J*_L_ is the moment of inertia of the RBC with the longitudinal axis as the reference axis. Assuming the RBC as a cylinder then gives *J*_L_ = *m*_RBC_ × *R*^2^/2. The unknown in Eq. 7 is the angular wall shear stress *f_AN_*, which will be estimated from the following DPD simulation results of case H.

A small patch of the RBC and endothelial cell interface was simulated using the DPD method. To find out whether or not the motion of EG can drive RBCs, water was included in the DPD simulation, but no flow was imposed on water. Although this is not a realistic physiological condition, the main purpose to isolate the effect of EG motion by taking advantage of the DPD simulation method. Considering the simulation domain is small, the curvature in Fig. 5 *b* can be neglected. Thus, the longitudinal and angular cases can share the same DPD model and results. In light of this, case H was established as shown in Fig. 5 *c*. Two arrays of glycocalyx chains were included: the upper array with three chains represents glycocalyx on the RBC side, and the lower array with five chains represents glycocalyx on the endothelial cell side. The five lower chains were first equilibrated in water (not shown in the figure for clarity), and then an initial velocity was exerted on the tips of the lower five chains as an activation of their dynamic motion at the very beginning of the simulation (from *t* = ∼0–5*τ*). Afterward, the simulation lasted for another 1595*τ* without external velocity input.

The velocity and shear rate distributions were calculated and averaged for the last 400*τ*, as shown in Fig. 5 *d*. The shear stress *f* can be calculated by *f* = *γδv*_*x*_/*δy*, with *γ* being the dynamic viscosity of water and *δv*_*x*_/*δy* the shear rate of the near-field flow. The shear stress that mainly contributes to the propulsion is from the region near the RBC surface, i.e., the region between *y* = 25*r*_c_ and *y* = 30*r*_c_. The time evolution for shear stress in this near-wall region is shown in Fig. 5 *e*. As suggested in Fig. 5, *d* (*right*) and *e*, the *δv*_*x*_/*δy* value from *y* = 25*r*_c_ to *y* = 30*r*_c_ was oscillating around zero, and the corresponding shear stress is weak. Therefore, the propulsion to RBCs from the EG is miniscule in both longitudinal and angular directions. Notably, in Fig. 5 *d* (*left*), the negative velocities of water during the time period of last 400*τ* imply that the RBCs are slowed down slightly when EG chains oscillate to the equilibrated positions.

### Some corollaries of described axisymmetrical versus nonaxisymmetrical layout interactions

**Figure 6:**
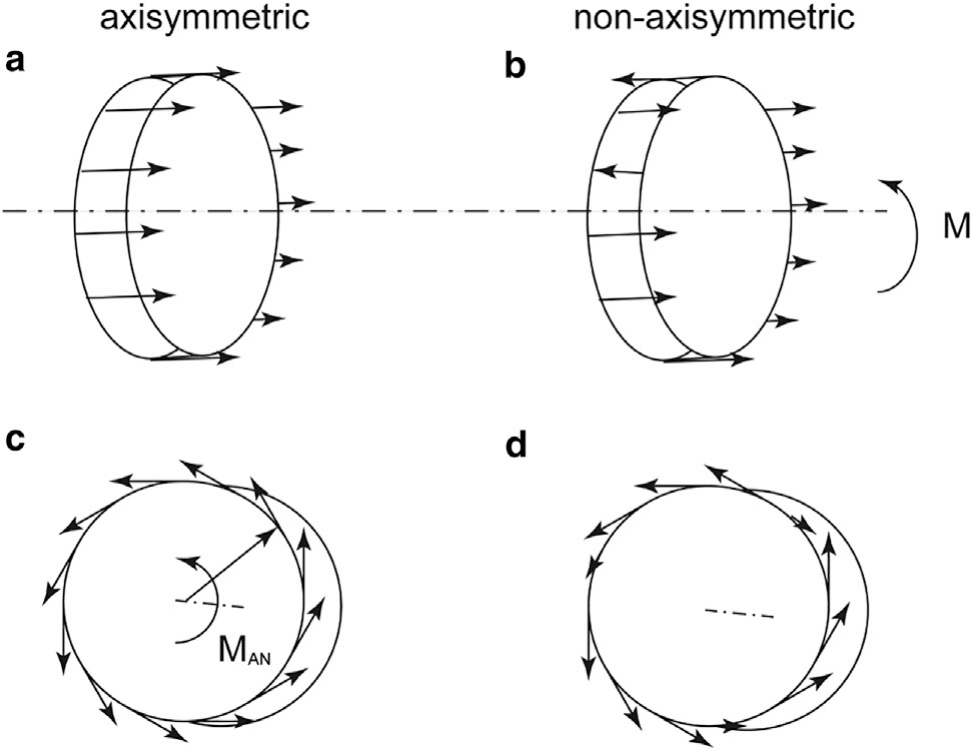
Force distribution with and without the “axisymmetry” assumption. (*a*) Force distributions under the assumption of axisymmetry. (*b*) Force distribution changes when the glycocalyx chains are nonaxisymmetrically distributed. (*c*) Shear stress distributions under the assumption of axisymmetry. (*d*) Shear stress distribution changes when the glycocalyx chains are nonaxisymmetrically distributed.

In EG as a propulsor for RBC movement, an axisymmetric layout of the glycocalyx was assumed. The influence of the nonaxisymmetric layout is to be discussed here. In the longitudinal direction, if the EG is not distributed in an axisymmetric pattern (e.g., variation in chain length or chain layout), the force would not be evenly distributed on the lateral outline of the RBCs (as shown in Fig. 6 *b*). As a result, the total force of *F*_L_ is more likely to decrease because some forces are negating each other. Meanwhile, the imbalanced force distribution would generate a moment of momentum which could potentially cause the rotation of RBCs along the radius. Similarly, the nonaxisymmetric layout in the angular direction would mitigate the aggregate angular force, as the orientations of some local shear stresses may be inverted (Fig. 6 *d*). Therefore, the propulsion from the EG would be further weakened.

In EG as a propulsor for RBC movement and Some corollaries of described axisymmetrical versus nonaxisymmetrical layout interactions, the possibility of the EG driving RBC movement was explored. The interaction between RBCs and EG develops platform for RBCs and the EG to communicate, and the findings obtained in these two sections provide an angle to understand the cross talk between the EG and RBC glycocalyx, as discussed below.

## Discussion

Cell experiments, DPD, and MD are all effective methods to study the EG behavior responding to surrounding changes but focus on different scales and aspects of EG problems. Compared with DPD and MD, results from cell experiments are more comprehensive and can be both biophysical and biochemical. MD and DPD are like in silico experiments, which provide temporally resolved, full-field data. MD, which is capable of tracing the trajectories of individual atoms, has the highest resolution among the three. It usually requires a gigantic amount of computational resources. As such, MD is normally used to study the dynamics of a local structure of the cell on nanoscales, like a small patch of lipid membrane with a few EG HS chains. MD can reproduce chemical reactions when proper force field parameters are available. DPD can be regarded as a coarse-grained MD that can reach much larger time and length scales. The computational resources required by DPD can be one or two orders lower (i.e., ∼0.1–0.01) than that of MD.

Herein, we exploited the DPD techniques to interrogate two- and three-compartment models of the EG and flow with or without the RBC glycocalyx, as each of them varies mimicking diverse physiological and disease conditions. Refined studies of these compartments are not amenable to experimental probing; thus, we sought to obtain insights through mathematical modeling. Previous analysis using a single Eulerian computational framework; (39) investigated the interactions between smooth RBCs (without the RBC glycocalyx) and EGs and revealed the possibility of the endothelial cell wall undulation, glycocalyx compression and repulsion, and temporal variations in the flow and shear stress. In our case, not only has the model been expanded to incorporate RBC glycocalyx, but we have also utilized subtle advantages of the DPD approach (see Materials and methods). Our expectation is that such an analysis may shed light on functional properties of the EG in damaged conditions due to pathological processes.

Damage to the glycocalyx has been described in multitude of pathologic conditions (5,6,40, 41, 42). It leads to a plethora of vascular pathologies (43,44). For example, activation of sheddases, heparanase, or hyalurinidase “prunes” defined components of the EG and results in impaired microcirculation (1, 2, 3). Indeed, as reported in case D (the short-chain case) of this study, the EG chains quickly achieve new equilibrium (Fig. 2*, a and b*), and the new equilibrated state can alter the force distribution of the actin cortical web (ACW), thereby inducing mechanotransduction (4). Chain shedding can also contribute to the loss of EG thickness. In Fig. 4, the flow tends to move following a similar direction to the shedding chain. The velocity projection on the endothelial cell surface is indeed reduced because of the upwards trend, and a reduced wall shear stress on the endothelial cell surface can be expected. Consequently, the mechanotransduction will be impaired.

**Figure 7:**
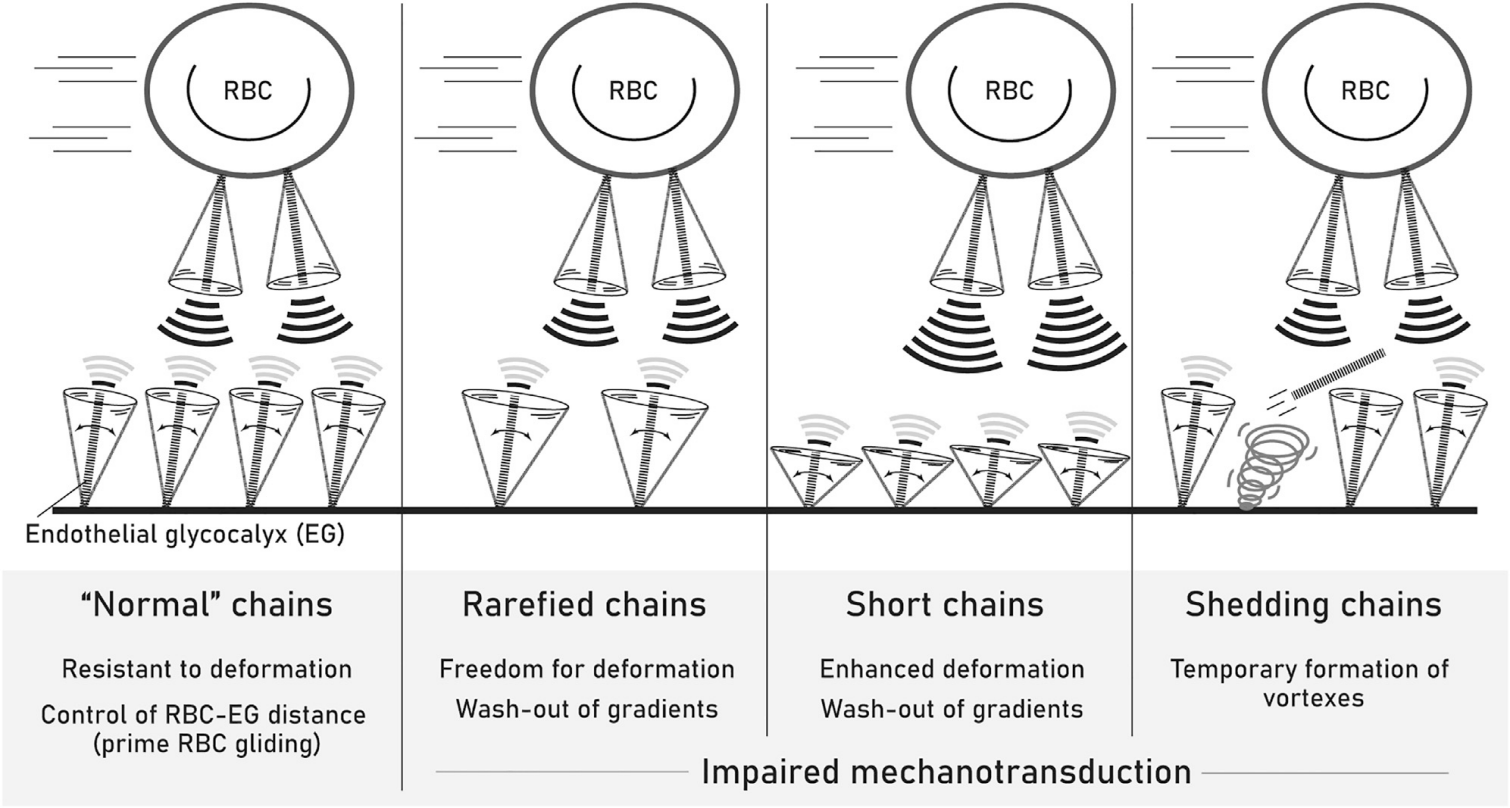
A schematic summary of DPD findings in the three-compartment model and extrapolations to possible functional consequences of perturbed flow, glycocalyx properties, and its shedding.

As reported here and schematically summarized in Fig. 7, the rarefaction of glycocalyx chains magnifies the single-chain deformation, and the mechanical signal via a single chain is enhanced. As shown in Fig. 2 *c*, the RMSD value of case C, which has a less compact EG layout, is ∼4.4 at the end of simulation and is ∼30% higher than that of the “normal” case (case A) with a value of ∼3.3. However, the mechanotransduction is the aggregate force on the ACW, which comprises the number of EG chains. From case A to case C, the number of chains is reduced by 50% (>30%), and the overall mechanical forces exerted on the ACW is weakened. Furthermore, as shown in Fig. 3, the chaos primed by the compact layout of glycocalyx chains, recreating physiological state, intensifies the interaction between the EG and flow, which actually benefits mechanotransduction. Otherwise, if most of the EG chains (mainly HS chains) were lost, the mechanotransducton by the EG would be seriously compromised as documented in a host of studies (45, 46, 47), with bioavailable NO deficiency and endothelial dysfunction ensuing (44).

EG also exerts an upward flow directed to the vascular lumen, as shown in Fig. 4. In Fig. 4, when EG chains shed, upward flow velocities are observed to increase. The upward flow thus may account for the washout of electrolyte and oncotic gradients normally maintained in EG, thereby disturbing local ion distribution, osmotic pressure, and ion permeability. The upward transport of sodium ions by shedding chains was also revealed in our previous MD study (22). Meanwhile, the reduction in EG dimension due to the trimming or shedding of HS chains further increases endothelial permeability and adhesion of leukocytes, thereby leading to proinflammatory vascular sites and atherosclerotic sequelae (48).

Indeed, the highly dynamic movement of EG chains primes changes in the surrounding flow field and allows frequent and varying fluid-structure interactions between the EG and surrounding flow. The active interactions further empower the EG to cross talk with circulating or surrounding cells. As documented in Oberleithner’s study, a positive correlation between the RBC and endothelial cell surface properties was observed, and the affected EG could lead to the shedding of RBC glycocalyx and vice versa; it is thus concluded that deranged glycocalyx of vascular endothelium leaves fingerprints on the surface of erythrocytes (15). This finding can be interpreted by these DPD results. Any loss or local impairment of EG would disturb the surrounding flow field (as shown in Fig. 4) and shear stress distribution. The local disturbance then induces force-torque imbalance to the RBC surfaces (as suggested in Some corollaries of described axisymmetrical versus nonaxisymmetrical layout interactions), altering the morphology of the RBC glycocalyx. In Fig. 2 *f*, weaker deformation of EG chains is observed when the RBC glycocalyx chain sheds, which means weaker response of the EG ensues and weakened mechanotransduction is expected. In other words, the shedding of RBC glycocalyx chains leads to the impairment of EG mechanotransduction—the main functionality of the EG. The force change in response to the EG density variation was also recorded in a previous MD study (49), in which the force to overcome the mechanical resistance of the EG was evaluated.

The importance of transition from laminar to turbulent flow or the appearance of oscillating flow in perturbations of microvascular homeostasis is hard to overestimate. Chien’s laboratory has demonstrated that oscillatory shear stress leads to inhibition of translation of peroxisome proliferators-activated receptor-*α* and enhanced expression of vascular cell adhesion molecule 1 and monocyte chemotactic protein 1 (50). In addition, whereas laminar shear stress increases endothelial level of sirtuin 1, oscillatory shear stress has an opposite effect on sirtuin-1, thus promoting inactivation of endothelial nitric oxide synthase (51). Endothelial cells subjected to oscillatory shear stress, in addition to the reduced bioavailable nitric oxide, acquire a proinflammatory phenotype with increased monocyte adhesion (52). Furthermore, oscillatory shear stress has been implicated in downregulation of antioxidative peroxiredoxins (53) and induction of mitochondrial superoxide (54). These multiple effects of nonlaminar flow and oscillating shear stress could be responsible for developing endothelial cell activation and dysfunction in the course of disease.

## Conclusions

In summary, the RBC glycocalyx and EG interactions and their interplay with flow are investigated via a DPD simulation method for the first time, to our knowledge. The research featuring interrogation by DPD of a three-compartment system consisting of RBC glycocalyx, EG, and flow offers extrapolations toward the integrative nature of the microcirculation and its derangements. This study reveals that the circulating RBCs drive the deformation of the EG via the near-field flow and the EG, in turn, exerts marginal propulsive force on RBCs. The EG deformation is sensitive to the chain layout, chain length, bending stiffness, RBC-EC distance, and RBC velocities. The disturbance to the flow by varying EG layout and shedding chains is also quantified. All these findings (summarized in Fig. 7) in the context of vascular physiology facilitate deeper appreciation of the roles played by impairments of glycocalyx and flow, respectively, in causing disorders of mechanotransduction and endothelial permeability under idealized conditions.

## Author contributions

X.Z.J. performed the research, analyzed data, and wrote the manuscript draft. M.S.G. designed the research, revised the manuscript, and supervised the project. K.H.L. revised the manuscript and supervised the project.
